# Regular nutritional monitoring in pediatric cancer patients undergoing radiotherapy: a prospective observational pilot study

**DOI:** 10.3389/fonc.2026.1752703

**Published:** 2026-03-12

**Authors:** Dilek Gül, Feyzanur Ekşi Özdaş, Nurşah Eker, Zerrin Özgen, Engin Tutar, Beste Melek Atasoy

**Affiliations:** 1 Radiation Oncology Clinic, Marmara University Pendik Education and Research Hospital, Istanbul, Türkiye; 2 Department of Nutrition and Dietetics, Marmara University Pendik Education and Research Hospital, Istanbul, Türkiye; 3 Department of Pediatrics, Division of Oncology and Hematology, Marmara University School of Medicine, Istanbul, Türkiye; 4 Department of Radiation Oncology, Marmara University School of Medicine, Istanbul, Türkiye; 5 Department of Pediatrics, Division of Gastroenterology, Marmara University School of Medicine, Istanbul, Türkiye

**Keywords:** caregiver support quality, clinical outcome, nutritional monitoring, pediatric cancer, quality of life, radiotherapy

## Abstract

**Objective:**

This pilot study aimed to explore the possible effects of regular, ongoing nutritional monitoring on dietary status, treatment results, and quality of life in pediatric cancer patients receiving radiotherapy. The study also sought to assess the potential role of caregiver nutritional support during treatment.

**Materials and methods:**

Anthropometric measures, including weight, height, body mass index z-score (BMI), and mid-upper arm circumference (MUAC), were recorded for 15 patients. Individual nutritional plans, the quality of caregiver-provided nutritional support, and inflammatory parameters were assessed weekly during radiotherapy. Quality of life was measured using a validated questionnaire at the beginning and end of radiotherapy.

**Results:**

More than one-third of patients were malnourished prior to radiotherapy. A decline was observed, especially in the fourth week of radiotherapy, for weight (p = 0.016), BMI (p = 0.013), and MUAC (p = 0.081). The albumin-to-globulin ratio (AGR) (p = 0.004) and platelet–lymphocyte ratio (PLR) (p = 0.013) peaked at the same time. Caregiver nutritional support scores (p = 0.026) and quality-of-life scores (p = 0.037) were lower in patients who experienced weight loss during radiotherapy. The median follow-up period was 13 months (range 9–16 months). Progression-free survival showed a non-significant trend in patients without weight loss [85.7% (95% CI, 42–99%) vs. 70% (95% CI, 35–93%), p = 0.590] and was significantly better in patients who began radiotherapy without malnutrition [100% (95% CI, 66–100%) vs. 50% (95% CI, 12–88%), p = 0.019].

**Conclusion:**

Our initial findings suggest that regular nutritional monitoring may be a part of treatment plans, not just supportive care, for pediatric patients undergoing radiotherapy. Further research involving larger patient populations should be conducted to establish high-quality evidence on this topic.

## 1 Introduction

Radiotherapy is an important treatment option for various pediatric solid and hematological cancers. Each year, many children receive radiotherapy for either curative or palliative reasons (1–5). Modern radiotherapy techniques can improve survival rates and enable more precise targeting of the disease, protecting healthy tissues (6–12). However, despite these advances, acute side effects caused by radiation therapy remain significant concerns in pediatric patients (13–16). These side effects are primarily determined by the dose and treatment area, often affecting normal tissues within the irradiated region. Additionally, systemic effects like changes in nutritional parameters and weight loss can worsen dietary deficits caused by cancer in patients undergoing radiotherapy (17).

Malnutrition generally leads to reduced treatment effectiveness, weakened immune function, lower quality of life, increased morbidity, and higher healthcare costs (17). At diagnosis, malnutrition is present in 50% of patients with solid tumors. This reflects major disturbances in body composition and energy balance, and malnutrition rates in some populations can reach 80% when more sensitive anthropometric measures are used (17). Radiation therapy can adversely affect dietary intake, gastrointestinal absorption, and whole−body metabolism through several mechanisms. Locoregional irradiation to the head and neck or upper aerodigestive tract commonly causes mucositis, dysphagia, odynophagia, taste alterations, xerostomia, and nausea, all of which reduce oral intake and contribute to acute weight loss and micronutrient deficiencies. When the abdomen or pelvis is irradiated, radiation enteritis, altered gastric emptying, and changes in intestinal motility and permeability can impair the absorption of macronutrients and bile acids, leading to diarrhea, steatorrhea, and additional nutrient losses. Beyond these local effects, radiotherapy increases oxidative stress and disrupts mitochondrial function and key pathways of glucose, lipid, and amino−acid metabolism, thereby promoting hypermetabolism, muscle catabolism, and unfavorable changes in body composition. These treatment−related metabolic perturbations frequently coexist with cancer−associated inflammation, which further accelerates protein and lipid breakdown and aggravates malnutrition (18). Multimodal treatments such as surgery and chemotherapy may worsen malnutrition due to their adverse side effects. The combined use of anticancer therapies can raise the frequency and severity of malnutrition in childhood cancer patients, both as acute and late effects (19). This can also affect survivorship, indirectly impacting the well-being of childhood cancer survivors. These data indicate that nutritional impairment is not a marginal issue, but a frequent and often under-recognized comorbidity in pediatric radiotherapy.

Inflammation associated with cancer and treatment may contribute to malnutrition by increasing protein and lipid breakdown and altering body composition during and after treatment (19, 20). In recent years, inflammatory markers and their ratios have been studied in pediatric patients with solid cancer. Elevated ratios have been associated with poor prognosis and may help predict nutritional status in pediatric patients with brain tumors (20).

Nutritional support and well-being may positively influence clinical outcomes and quality of life (21, 22). Malnourished pediatric patients are more likely to experience severe treatment−related toxicities, infections, and interruptions or dose reductions of chemotherapy and radiotherapy, which may compromise local control and overall outcomes. Nutritional deficiencies have been linked to impaired functional status and markedly reduced health−related quality of life in patients receiving radiotherapy, particularly for head and neck and central nervous system tumors. These observations highlight the need to systematically identify and address nutritional problems before and during radiotherapy to optimize tolerance, minimize acute and late toxicities, support recovery, and potentially improve long−term survival in childhood cancer (21). Therefore, it is essential to optimize nutritional counseling and manage malnutrition by promptly initiating appropriate interventions and closely monitoring patients throughout radiotherapy.

In this prospective pilot study, we aimed to assess and report changes in anthropometric and nutritional parameters among pediatric cancer patients who were closely monitored and received regular nutritional counseling during radiotherapy. We also sought to explore whether this approach could influence treatment outcomes and quality of life.

## 2 Materials and methods

The study protocol was approved by the Marmara University School of Medicine Non-Interventional Clinical Research Ethics Committee (reference number 09.2023.406 and date 03.03.2023). Patients who received radiotherapy and provided informed consent participated in this study. The study was conducted in accordance with the principles of the Declaration of Helsinki. All patients, including their caregivers, were informed, and written consent was obtained before radiotherapy. Patients aged 8 years or older were included, as the Turkish validation of the quality-of-life questionnaire used in the study was limited to this age group. Demographic variables were collected from parents at the beginning of the study. The protocol was initially designed for a one-year period, with a planned sample size of 30 patients. However, due to unexpectedly slow recruitment, this study was terminated early, and we decided to report the preliminary results with a final sample of 15 patients.

### 2.1 Nutritional assessment and evaluation

#### 2.1.1 Anthropometric measurements

The study design is summarized in **Table 1**. Anthropometric measurements, including weight, height, and mid-upper arm circumference (MUAC), were recorded for each patient (23–25). Weight and height were measured using a calibrated electronic scale in child mode (Densi GL-150 Automatic Height Weight BMI Measurer, Istanbul, Türkiye), with children wearing minimal clothing, including no socks or shoes. The MUAC is the circumference of the right upper arm measured at the midpoint between the shoulder tip and elbow. The tape was placed perpendicular to the arm’s long axis, with the arm flexed to 90° (24). The MUAC-for-age z score was calculated as previously described (25, 26). Nutritional status was determined based on the Centers for Disease Control and Prevention (CDC) BMI-for-age indicators (5–19 years) (27).

**Table 1:** Study design and measures.

Study measures	Beginning of radiotherapy	During radiotherapy (weekly)	End of radiotherapy
Demographics*	+		
Nutritional Assessment	+	+	+
The STRONG-kids nutritional risk screening tool	+	+	+
Anthropometric measurements**	+	+	+
Body mass index-for-age z score	+	+	+
Diet counseling and prescribing	+	+	+
Laboratory tests***	+	+	+
Radiotherapy side effects evaluation^¶^	+	+	+
Patients Quality of Life Assessment^δ^	+		+
Caregiver evaluation			
Quality of nutritional support	+	+	+
The Hospital Anxiety and Depression Scale	+		+
The Multidimensional Scale of Perceived Social Support Scale	+		+

*Age, gender, date of birth, diagnosis, stage, previous treatments; parents’ characteristics like education, working status, number of children, social and economic status;**Weight (kg), height (cm), mid-upper arm circumference (MUAC) for age z score; ***Hemograms, total protein, and albumin; ^¶^According to Common Toxicity Criteria v5.0; ^δ^Pediatric Quality of Life Assessment tool.

#### 2.1.2 Biochemical and inflammatory parameters

All laboratory tests (hemoglobin, g/dL; total protein, g/L; albumin, g/L) were conducted weekly for each patient. The albumin-to-globulin ratio (AGR), neutrophil-lymphocyte ratio (NLR = the ratio of neutrophil count to lymphocyte count), platelet-lymphocyte ratio (PLR = the ratio of platelet counts to lymphocyte count), and systemic inflammatory index (SII = platelet count divided by NLR) were calculated as markers of inflammation (28).

#### 2.1.3 Nutritional risk screening

Nutritional risk was assessed using the Turkish version of the STRONG-Kids Nutritional Risk Screening Tool (29). This tool includes four scored questions covering the underlying disease, subjective clinical assessment, high-risk disease, food intake, losses (e.g., nausea and vomiting), and weight changes. The total score was determined on a scale from 0 to 5. Patients scoring 1–3 points were classified as low risk, while those with 4–5 points were classified as high risk at the time of assessment (30).

#### 2.1.4 Dietary counseling and evaluation

The nutritional evaluation began with dietary recording. Caregivers provided a three-day consecutive dietary food record to document nutritional intake at the start and end of radiotherapy. Then, the dietitian interviewed each caregiver and their child at the beginning of radiotherapy. The questions covered the children’s current eating habits, family behaviors, grocery shopping, food hygiene, storage, cooking methods, serving practices, and daily lunch and dinner routines. The interview was repeated weekly to monitor patients’ prescribed dietary intake and adherence to nutritional guidelines from the previous week; adherence was operationally defined as consuming at least 80% of the individually prescribed daily and protein targets, as estimated from the three-day record and caregiver report. During these visits, caregivers discussed reasons for non-compliance and any barriers to adherence (such as treatment-related symptoms, food aversions, or logistical constraints) were documented in the study forms. The radiation oncologist (DG) recorded each patient’s complaints and symptoms weekly during radiotherapy.

Personalized nutritional plans were created based on each patient’s weekly condition. The Schofield equation was used to estimate basal metabolic rates (31). Protein intake was adjusted to 15–20% of total daily energy needs. During weekly assessments, oral nutrition supplements (ONS) were prescribed when patients were unable to meet their daily energy and protein requirements through food intake, in accordance with guidelines (17). Patients who were unable to meet their daily energy needs and continued to lose weight received immediate nutritional support through ONS before starting radiotherapy.

#### 2.1.5 Patient quality of life evaluation

All patients completed the Turkish version of the Pediatric Quality of Life Assessment forms at the beginning and end of radiotherapy (32). The twenty-three items cover physical (8), emotional (5), social (5), and school functioning (5). Patients were asked to consider the past month for each question when responding to the scale items. A five-point response scale was used across child self-report: 0 = never a problem, 1 = rarely a problem, 2 = sometimes a problem, 3 = often a problem, and 4 = almost always a problem. Items are reverse-scored and linearly converted to a 0–100 scale (0 = 100, 1 = 75, 2 = 50, 3 = 25, 4 = 0), with higher scores indicating better quality of life (33).

#### 2.1.6 Caregiver evaluation

The caregivers were assessed using the Turkish versions of the Hospital Anxiety and Depression Scale (HADS) and the Multidimensional Scale of Perceived Social Support (MSPSS) (34, 35). The HADS is a 14-item self-report rating scale scored on a 4-point Likert scale (0–3). It is designed to measure anxiety and depression, with seven items for each subscale. The overall score is the sum of all 14 items, while each subscale score is the sum of its seven items (range, 0–21) (36). The MSPSS is a 12-item self-report instrument that evaluates perceived social support from family, friends, and significant others. Respondents rate each item on a 7-point Likert scale (strongly disagree to strongly agree) (37).

A radiation oncologist and a dietitian evaluated caregivers’ quality of nutritional support weekly using a previously developed questionnaire (38). The seven questions addressed caregivers’ concern for patients, attentiveness to patient care, adherence to nutrition recommendations, interest in and desire for nutritional support, understanding of nutrition’s importance, ability to be creative and playful, and persuasive power over the patient regarding nutrition. The total score was calculated from responses, with scores assigned as follows: unconcerned = 1, some concern = 1, adequate = 3, very good = 4, and excellent = 5. The highest possible score was 35.

### 2.2 Radiochemotherapy

All patients were simulated on a computed tomography simulator, and appropriate immobilization was prepared for each patient. Treatment planning was conducted using Volumetric Modulated Arc Therapy (VMAT) in the Monaco Treatment Planning System, version 5.11. All patients received radiotherapy with a linear accelerator delivering 6 MV photons (Elekta Synergy, Crawley, UK). Daily treatments involved image-guided radiation therapy using kV portal imaging. Concomitant chemotherapy was managed by a pediatric oncologist (NE). Concurrent temozolomide was prescribed at 90 mg/m2 for 42 days for patients with high-grade glial tumors (n=7). Patients with medulloblastoma received weekly vincristine at 1.5 mg/m2 (maximum dose 2 mg) during radiotherapy (n=2). One patient with Ewing sarcoma continued chemotherapy during radiotherapy without Adriamycin (n=1). Side effects were recorded according to the Common Toxicity Criteria version 5.0.39 (39).

### 2.3 Statistical analyses

Statistical analyses were conducted using IBM SPSS Statistics version 20.0 (IBM Corp., Armonk, NY, USA). For descriptive purposes, numerical variables were summarized using either the mean, standard deviation, or median. The results are presented as mean ± 2 standard deviations. The study population was analyzed weekly for anthropometric measurements, laboratory results, and the quality of caregiver-provided nutritional support. Weight, BMI, and MUAC measurements were included in the analysis. Total radiation dose, planning target volume, chemotherapy, and side effects were also considered during the comparison analysis. The Shapiro–Wilk test was used to assess normality; skewness and kurtosis values within the range of −1.5 to +1.5 indicate a normal distribution. Therefore, a paired samples t-test was used to compare weekly changes in the mean of each variable.

Progression-free survival (PFS) was defined as the time from the start of radiochemotherapy to the first occurrence of either clinical or radiological progression. Survival curves were estimated using the Kaplan–Meier method, and between-group comparisons were conducted using the log-rank test. A two-sided p-value less than 0.05 was considered statistically significant.

## 3 Results

The characteristics, diseases, and treatments of the 15 participants are summarized in **Table 2**. Most were diagnosed with central nervous system tumors (n = 11). The median radiotherapy dose was 54 Gy (range, 30–60 Gy), delivered over a median of 7 weeks (range, 4–7 weeks). All patients completed radiotherapy and concurrent chemotherapy as planned. No clinical or radiological progression was observed during radiotherapy. None of the patients received prophylactic corticosteroids. However, two patients with brain tumors required short-term steroids for radiation-induced intracranial edema (headache) occurring between the 3rd and 5th weeks of radiotherapy. During the analysis, one patient was assigned to the weight-loss group and the other to the no-weight-loss group.

**Table 2:** Characteristics of study population, disease, and treatment.

Characteristics	
Gender (Male: Female) in number	(10:5)
	Median (IQR)
Age/years	13 (8-16)
Total radiotherapy dose (Gy)	54 (30-60)
Planning target volume (cc)	
Phase 1	273.77 (38.64-2642.28)
Phase 2	178.97 (38.45-374.95)
Radiotherapy duration (week)	7 (4-7)
	n (%)
Diagnosis	
Brain tumor	11 (73.3)
High grade glial	7 (46.6)
Medulloblastoma	3 (20)
Low grade glial	1 (6.7)
Locally advanced nasopharynx cancer	2 (13.3)
Early-stage Hodgkin lymphoma	1 (6.7)
Ewing’s sarcoma	1 (6.7)
Radiotherapy field	
Cranial	7 (46.6)
Craniospinal	3 (20)
Spinal	2 (13.3)
Nasopharynx and cervical lymph nodes	2 (13.3)
Mediastinal lymph nodes	1 (6.7)
Chemotherapy	
Concomitant	11 (73.3)
Neoadjuvant	4 (26.6)
Grade 3-4 side effects related to nutrition	
Loss of appetite	12 (80)
Nausea/vomiting	7 (46.6)
Dysphagia	5 (30)
Taste alteration	4 (26.6)
Xerostomia	3 (20)
Diarrhea/constipation	3 (20)
Oropharyngeal mucositis	2 (13.3)
Patients’ BMI-for-age z scores (5-19 years) at the beginning of radiotherapy	
Obesity: >+2SD	0
Overweight: >+1SD	3 (20)
+1 SD -1 SD	6 (40)
Thinness: <-2SD	6 (40)
Severe thinness: <-3SD	0

The most significant toxicities that may affect nutritional status include loss of appetite (n = 12), nausea/vomiting (n = 7), dysphagia (n = 5), and taste alteration (n = 4). These side effects may also be associated with concurrent chemotherapy.

The median interval between diagnosis and radiotherapy was 3 months (range, 2–8 months). At diagnosis, six (40%) patients were already malnourished, with a median BMI of 0.31 (range, -2.41 to 1.68). Weekly anthropometric, laboratory, and inflammatory measurements are shown in **Table 3**. Weight, BMI, and MUAC declined gradually over the first 4 weeks, with the largest mean decreases at week 4 (weight, p = 0.016; BMI, p = 0.013), followed by relative stabilization thereafter. During the same week, 80% of children screened for nutritional risk on STRONGkids, and inflammatory parameters peaked in parallel, with significant increases in AGR (p = 0.004) and PLR (p = 0.013).

**Table 3:** Longitudinal study measures across radiotherapy weeks.

	Week 1	Week 2	Week 3	Week 4	Week 5	Week 6	Week 7
	Mean ± SD (minimum-maximum)						
Screening test score							
STRONG-kids score	3.0 ± 1.25 (2-5)	3.4 ± 1.12 (2-5)	3.8 ± 1.18 (2-5)	4.0 ± 1.27 (2-5)	3.9 ± 1.12 (2-5)	3.4 ± 1.25 (2-5)	3.4 ± 1.24 (2-5)
Anthropometric measures							
Weight (kg)	46.12 ± 17.3 (20.8-78.7)	45.75 ± 17.32 (22.5-78.1)	45.21 ± 16.96 (22.6-77.7)	44.54 ± 16.91 (21.0-77.8)	44.10 ± 16.79 (21.0-78)	44.27 ± 16.79 (21.4-79.2)	44.48 ± 16.62 (21.8-79)
MUAC for age z scores	-0.415 (--2.10-0.65)	-0.537 (-2.11-0.64)	-0.510 (-2.11-0.64)	-0.532 (-1.89-0.69)	-0.543 (-1.89-0.66)	-0.537 (-1.79-0.65)	-0.440 (-2.19-0.66)
Body mass index-for-age z score	-0.10 ± 1.24 (-2.15-1.68)	-0.21 ± 1.20 (-2.28-1.70)	-0.20 ± 1.31 (-2.82-1.66)	-0.39 ± 1.46 (-2.82-1.72)	-0.32 ± 1.4 (-2.74-1.71)	-0.45 ± 1.32 (-2.34-1.45)	-0.51 ± 1.27 (-2.33-1.72)
Laboratory measures							
Hemoglobin (g/dL)	12.3 ± 1.87 (8.4-14.6)	12.3 ± 1.54 (8.6-15.0)	12.4 ± 1.62 (8.6-15.5)	12.1 ± 1.82 (8.3-15.4)	11.9 ± 2.10 (8.4-15.6)	11.5 ± 2.16 (8.4-15.6)	11.9 ± 2.28 (9.0-16.5)
Total protein (g/L)	71.60 ± 5.80 (59-80)	70.39 ± 6.12 (57-80)	71.03 ± 5.78 (60-80)	69.96 ± 5.83 (58-79)	69.09 ± 6.69 (54-78)	68.52 ± 5.64 (56-78)	70.57 ± 5.54 (61-79)
Albumin (g/L)	46.4 ± 3.66 (37-52)	46.42 ± 3.69 (39-52)	47.50 ± 3.0 (42-51)	47.07 ± 3.31 (42-54)	45.71 ± 4.82 (33-53)	44.5 ± 4.75 (32-52)	45.70 ± 3.11 (40-53)
Inflammatory markers							
AGR	1.86 ± 0.30 (1.50-2.47)	1.93 ± 0.28 (1.52-2.49)	2.07 ± 0.37 (1.50-3.13)	2.11 ± 0.34 (1.54-2.62)	2.05 ± 0.54 (1.43-3.26)	1.88 ± 0.32 (1.33-2.36)	1.89 ± 0.53 (1.03-2.94)
NLR	4.26 ± 4.78 (1-14)	3.07 ± 2.33 (1-9)	3.50 ± 3.36 (1-12)	6.50 ± 8.32 (1-30)	5.60 ± 4.33 (2-19)	9.64 ± 17.67 (1-70)	6.0 ± 7.68 (1-32)
PLR	208.03 ± 204.20 (49.1-710)	281.79 ± 196.06 (80.8-640)	278.62 ± 177.83 (71.7-606.6)	315.73 ± 257.48 (84.7-1010)	326.19 ± 251.79 (116.6-960)	369.45 ± 332.37 (103.3-1045)	315.09 ± 256.92 (94.4-1115)
SII	856.46 ± 951.13 (142.5-4264)	856.38 ± 835.95 (145.0-3264)	732.95 ± 415.83 (165.0-1639.0)	1088.39 ± 1067.07 (284.6-4307)	1133.0 ± 688.97 (335-2620)	1193.20 ± 1090.30 (244.8-4060)	1011.24 ± 622.77 (256.6-2140.8)

MUAC, The mid-upper arm circumference; AGR, albumin globulin ratio; NLR, neutrophil-lymphocyte ratio; PLR, platelet lymphocyte ratio; SII, systemic inflammatory index; The difference between the values of week three and week 4 for weight, p=0.016, body mass index-for-age z score p=0.016 and MUAC (p=0.081); of week one and week 4 for weight, p=0.008 and body mass index-for-age z score p=0.013; albumin globulin ratio p=0.004 and platelet lymphocyte ratio p=0.0013; for week one and week 7 for weight p=0.038.

At the start of treatment, the STRONGkids score was ≥3, indicating a risk of mild malnutrition in 60% of patients. By the end, this rate had risen to 73.3%. In this cohort of 15 children, eight patients (53.3%; 95% CI, approximately 28–79%) lost weight during treatment, with a mean weight loss of 3.49 ± 2.58 kg, corresponding to a mean relative loss of 6.9% (range, 1.55–15.35%).

The remaining patients neither lost nor gained weight (mean, 0.52 ± 0.24 kg). From week 1 to week 7, the mean BMI (–0.15 ± 1.31 vs. –0.74 ± 1.29) and MUAC (–0.36 ± 0.95 vs. –0.66 ± 0.62) were lower in patients who lost weight than in those who did not. No significant differences were observed between patients who lost weight and those who did not with respect to diagnosis/disease site, radiation dose, planning target volume, chemotherapy, or grade 3–4 side effects that could affect nutritional status. Similarly, the average inflammatory parameters (AGR, NLR, PLR, and SII) showed no significant difference between patients who lost weight or experienced poor weight gain and those from previous weeks of radiotherapy (**Table 4**).

**Table 4:** Comparison of treatment characteristics and study measures according to weight loss during radiotherapy.

	Patients		p
	Weight loss n=8	No Weight loss n=7	
	Mean ± SD		
Treatment and disease details			
Radiotherapy dose (Gy)	55.12 ± 5.4	52.28 ± 10.22	0.529
Planning target volume -Phase 1 (cc)	654.69 ± 855.88	705.56 ± 939.28	0.915
Planning target volume- Phase 2 (cc)	205.25 ± 103.37	163.88 ± 90.05	0.542
	n (%)		
Early stage	2	2	0.887
Locally advanced stage	6	5
Concomitant chemotherapy	7 (87.5)	4 (57.1)	0.230
Neoadjuvant chemotherapy	3 (37.5)	1 (14.3)	0.336
Grade 3-4 side effects related to nutrition			
Loss of appetite	8 (100)	4 (57.1)	0.078
Nausea/vomiting	4 (50)	3 (42.9)	0.800
Dysphagia	4 (50)	1 (14.3)	0.156
Taste alteration	3 (37.5)	1 (14.3)	0.336
Xerostomia	3 (37.5)	0	0.08
Diarrhea/constipation	3 (37.5)	0	0.08
Oral-pharyngeal mucositis	2 (25)	0	0.170
Score of caregiver’s nutritional support quality			
Week-1	25.25 ± 3.41	27.0 ± 5.39	0.454
Week-2	24.37 ± 3.11	27.57 ± 6.24	0.222
Week-3	26.50 ± 4.27	27.28 ± 6.01	0.773
Week-4	24.37 ± 4.24	27.42 ± 4.19	0.756
Week-5	23.87 ± 3.60	28.85 ± 4.05	**0.026**
Week-6	25.12 ± 4.05	28.10 ± 4.41	0.191
Week-7	25.00 ± 4.24	29.42 ± 3.25	**0.043**
Inflammatory markers			
AGR	1.90 ± 0.34	2.00 ± 0.35	0.593
NLR	6.39 ± 6.74	3.71 ± 2.60	0.326
PLR	275.74 ± 136.68	333.24 ± 280.89	0.635
SII	843.98 ± 883.52	870.72 ± 1095.30	0.960

AGR, albumin globulin ratio; NLR, neutrophil-lymphocyte ratio; PLR, platelet lymphocyte ratio; SII, systemic inflammatory index.

The bold values are the significant p values.

The caregivers’ nutrition support scores did not change significantly over the seven-week period, with median values remaining in the ‘adequate’ to ‘very good’ range. However, the lowest average score was recorded during the fourth week of radiotherapy (25.80 ± 4.36), coinciding with the peak in nutritional risk and inflammatory markers. Among children who lost weight, caregiver support scores were significantly lower than those of caregivers of children without weight loss at week 5 (mean difference, approximately 3 points; p = 0.026) and at the end of radiotherapy (p = 0.043) (**Table 4**). However, no significant differences in HAD scores among caregivers were found based on their children’s weight loss. Although not statistically significant, caregivers of children who experienced weight loss reported lower social support scores from family and friends at the end of radiotherapy.

**Figure 1:**
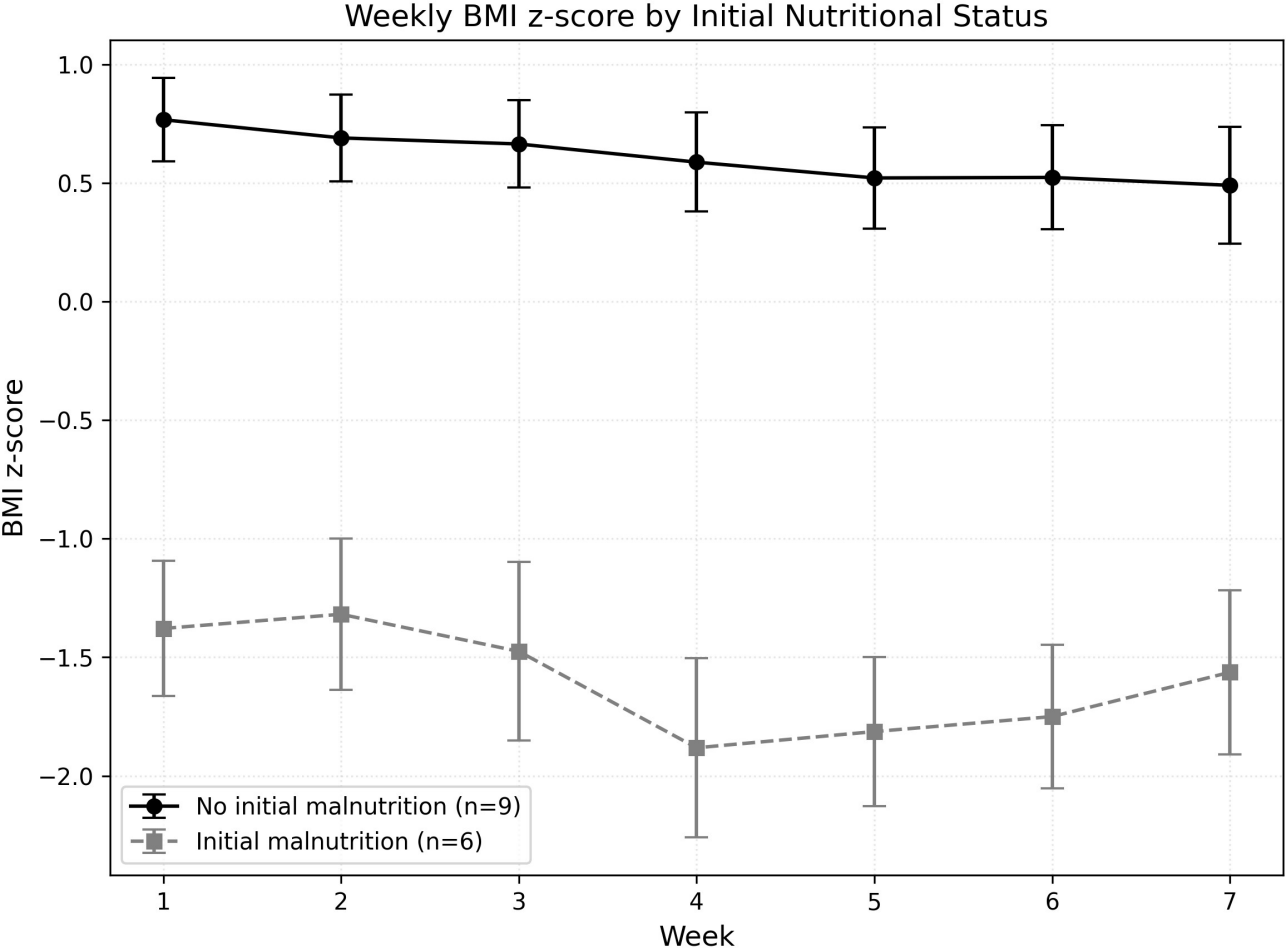
Weekly changes in BMI z−score according to initial nutritional status. Mean BMI z−scores (± standard error) are plotted for children without initial malnutrition (black circles, solid line; n = 9) and with initial malnutrition (gray squares, dashed line; n = 6) across weeks 1–7 of radiochemotherapy.

Changes in physical, emotional, social, and school-related quality of life did not differ significantly between patients based on weight loss during radiotherapy (**Table 5**). At the start of radiotherapy, six patients (40%) were already malnourished due to factors such as induction chemotherapy (n = 1), diencephalic syndrome (n = 1), or socioeconomic issues (n = 3). Despite intensive follow-up and support, these patients remained malnourished by the end of radiotherapy. **Figure 1** shows the weekly BMI for patients who were malnourished at the start of radiotherapy and those who were not. The BMI scores differed significantly each week of treatment (p = 0.001; p < 0.0001). Meanwhile, MUAC measurements showed no significant difference between the two groups.

**Table 5:** Patient quality of life assessment according to weight loss or baseline malnutrition.

Quality of life (scores)	Weight loss during radiotherapy				Malnutrition at the beginning of radiotherapy			
	(+) n=8	p	(-) n=7	p	(+) n=5	p	(-) n=10	p
	Mean ± SD		Mean ± SD					
General								
Beginning	63.58 ± 18.59	0.257	58.65 ± 24.41	0.197	52.46 ± 18.19	0.176	65.69 ± 21.55	0.769
End	59.21 ± 18.45		69.34 ± 13.75		63.54 ± 18.62		64.14 ± 16.72	
Physical								
Beginning	59.37 ± 17.91	0.955	62.49 ± 29.75	0.276	41.87 ± 17.75	**0.037**	70.30 ± 20.10	0.541
End	58.98 ± 13.40		70.98 ± 21.70		59.99 ± 6.01		66.87 ± 21.91	
Emotional								
Beginning	61.87 ± 25.34	0.627	66.66 ± 20.41	0.523	53.00 ± 18.90	0.215	70.00 ± 23.18	0.450
End	56.35 ± 23.10		72.14 ± 13.18		65.00 ± 22.92		62.22 ± 21.08	
Social								
Beginning	78.75 ± 24.31	0.531	76.42 ± 26.09	0.354	76.00 ± 30.29	0.118	78.50 ± 22.49	0.357
End	80.00 ± 23.75		87.14 ± 11.49		78.00 ± 25.14		86.00 ± 15.59	
School								
Beginning	54.37 ± 23.05	0.631	45.00 ± 37.68	0.502	39.00 ± 27.70	0.440	56.51 ± 29.68	0.582
End	48.12 ± 37.21		47.14 ± 25.47		51.00 ± 30.49		51.11 ± 30.49	

The bold values are the significant p values.

**Figure 2:**
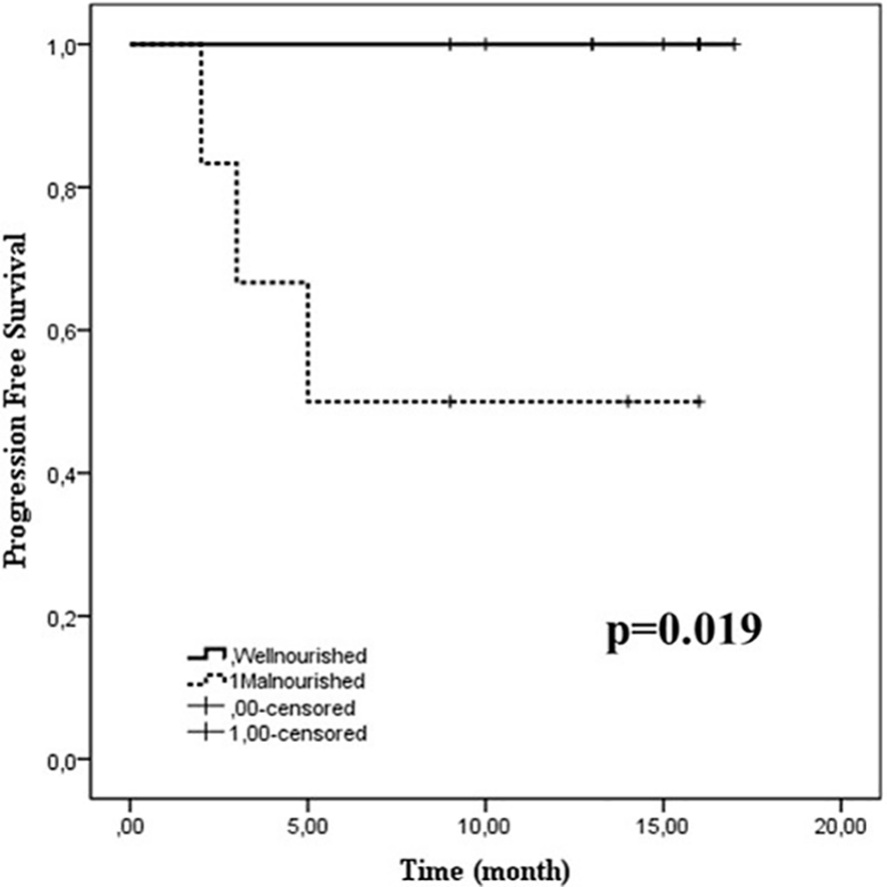
Kaplan-Meier curves of progression-free survival in pediatric patients stratified by baseline nutritional status. The solid line represents well-nourished patients, while the dotted line indicates malnourished patients. Tick marks denote censored cases. Well-nourished patients had significantly longer progression-free survival than malnourished patients (p = 0.019).

At the time of analysis, the median follow-up was 13 months (range, 9–16 months). Progression-free survival showed a non-significant trend in patients without weight loss [85.7% (95% CI, 42–99%) vs. 70% (95% CI, 35–93%), p = 0.590] and was significantly better in patients who began radiotherapy without malnutrition [100% (95% CI, 66–100%) vs. 50% (95% CI, 12–88%), p = 0.019] (**Figure 2**).

## 4 Discussion

We observed that radiotherapy may lead to deterioration in nutritional parameters in our cohort, as previously reported (18–20). Although the follow-up period was relatively short, nutritional deterioration may also impact survival outcomes in this study. Fabozzi et al. (40) proposed the A-B-C-D method, which includes anthropometric measurements (A), biochemical tests (B), clinical evaluation (C), and dietary intake (D) for nutritional screening in children with cancer. They recommended that patients undergoing intensive treatment or at high risk of malnutrition be evaluated at intervals of no more than 3–4 weeks. Other studies have highlighted the importance of early nutritional intervention, ideally managed by a nutrition-specific team (41, 42). The protocol used in this study was developed in accordance with these recommendations.

To our knowledge, this is the first study to longitudinally examine nutritional changes during radiotherapy, monitoring patients weekly from initiation to completion. Nutritional risk was assessed using the STRONGkids screening tool, with scores indicating increasing malnutrition risk as therapy continued. In our cohort, more than 50% of patients experienced weight loss during treatment, regardless of radiotherapy planning parameters (dose, volume, and field size). The highest risk for malnutrition occurred between the start and the midpoint of radiotherapy.

The weight loss group experienced more severe side effects during radiotherapy, as measured by intensity and grade. The most significant side effects were nausea and vomiting, which may be caused by concomitant chemotherapy. Patients may refuse dietary supplements, which can increase the severity of toxicities during systemic treatment. In our study, side effects may lead to weight loss in pediatric patients, as observed in adult patients with head and neck cancer.

For patients with head and neck cancer, close nutritional monitoring and support from a nutrition team may be essential during radiochemotherapy, as these patients face a high risk of malnutrition and its consequences (43). Similarly, a multidisciplinary nutritional intervention in pediatric patients may be started at the beginning of radiotherapy. Early intervention and nutritional education may help improve their quality of life (44).

Malnutrition is associated with poorer survival in pediatric patients with brain tumors (45). Survival was significantly worse at diagnosis or three months after diagnosis for patients who were malnourished than for those who were well-nourished at diagnosis. In our study, all patients who had malnutrition before radiotherapy were diagnosed with brain tumors. In addition, our patients, who had malnutrition at the time of diagnosis and started radiotherapy with weight loss, maintained this condition until the end of treatment.

In this cohort, the quality of caregivers’ nutritional support for children who experienced weight loss was significantly lower than for those who did not. Nutritional toxicities were not substantially different between patients with weight loss and those with weight gain in the study. Therefore, we believe that the role of caregivers in nutritional care during radiotherapy deserves further investigation.

Radiation therapy can be stressful for both patients and their families (46). Family-centered care, including caregiver support, may play a role during radiotherapy, especially in combating malnutrition. They express feelings of uncertainty about their children’s prognoses (47, 48). Patients undergoing radiotherapy have often been diagnosed and treated in surgery or chemotherapy clinics. Although they experience their first shock and subsequent symptoms, worries about the future persist.

Caregivers can become exhausted before and during prolonged treatments such as radiotherapy (49). In our sample, nutritional support scores declined steadily as treatment concluded. We also observed increased post-treatment anxiety, which might be linked to improvements in patients’ symptoms. Caregivers of patients experiencing weight loss showed slightly elevated anxiety levels. They also face weekly fatigue due to social issues, hospital stays, and financial concerns. Additionally, we found that MSPSS scores were lower among caregivers of patients with weight loss. These findings may suggest that lower caregiver engagement and perceived ability to support nutrition may be associated with poorer weight maintenance during treatment in this small cohort. Therefore, we believe that including caregiver support in treatment may improve outcomes. During the study, physicians noted that it is challenging for parents to feed their children, especially when they become physically exhausted by their child’s refusal to eat. Pediatric radiotherapy may involve preparing caregivers with empathetic, skilled staff who recognize the child’s suffering and intense emotions (49). A study reported that caregivers may become fatigued, struggle, or attempt to coerce their child to eat, as we observed (50).

In recent years, inflammatory markers have been studied as potential unfavorable prognostic indicators (51). In our study, these markers were elevated in patients with initial malnutrition. Additionally, PLR and AGR values significantly increased together in the fourth week, when weight loss peaked. Therefore, future research may evaluate the roles of these inexpensive, simple biomarkers alongside anthropometric measurements.

In summary, epidemiological data show that the nutritional status of children with cancer is highly heterogeneous, with both undernutrition and overnutrition frequently observed and substantial variation by tumor type, treatment modality, and treatment phase. Recent cohort studies report that the prevalence of undernutrition during therapy ranges from single−digit percentages to well over one−third of patients (52). Further studies may include inflammatory markers, nutritional status across various tumor types, socioeconomic context, and their influence on outcomes, treatment−related toxicity, and nutritional adherence.

### 4.1 Limitations

This is a prospective single-arm observational pilot study. Since having an observation arm without nutritional intervention is not ethically appropriate, there is no prospectively planned control arm. Additionally, to examine the effects of radiotherapy on nutrition, changes in nutritional parameters were compared, particularly at the beginning of radiotherapy. The absence of a control arm may preclude a clear separation of the effects of radiotherapy from those attributable to the nutritional counseling and ONS protocol and may limit causal inference regarding the specific contribution of nutritional support to changes in anthropometric, biochemical, inflammatory, and quality−of−life parameters.

The short follow-up period limits the ability to report long-term survival outcomes and overall survival rates. Additionally, the tumor sites in our study varied. Typically, radiotherapy clinics that are not designated as pediatric reference centers treat about 10–15% of all patients. Within this group, most diagnoses are for central nervous system (CNS) tumors; however, a heterogeneous diagnostic profile is inevitably present. Therefore, we believe that our pilot study may reflect daily clinical practice regarding the distribution of patient diagnoses. Meanwhile, future research may focus on homogeneous tumor groups with larger sample sizes to generate high-quality, evidence-based results.

The main limitation of our study is the small sample size. All radiotherapy clinics adhere to protocols for the treatment and monitoring of pediatric tumors, and nutritional evaluation is a standard component of clinical practice. However, its relevance and significance need further investigation, and obstacles to implementation must be clarified. Our goal is to emphasize the need for caution on this topic, and we believe further research in this area is necessary.

Given these limitations, it may be advisable to interpret the findings as hypothesis−generating and descriptive of outcomes under an integrated radiotherapy plus nutritional support program, rather than as definitive evidence of the isolated impact of the nutritional intervention.

In our cohort, we did not measure other anthropometric parameters of body composition, the distribution of muscle, fat, and bone mass. These could also be included in future research on pediatric patients.

## 5 Conclusion

Radiotherapy alone may increase the risk of malnutrition in pediatric cancer patients. Every patient undergoing radiotherapy is at risk of severe malnutrition by the end of treatment. This risk may be even higher in patients who are already malnourished before beginning radiotherapy. Therefore, nutritional monitoring and management may be recommended weekly, with a multidisciplinary team that includes caregivers, and integrated into primary cancer treatment. We believe that the impact of regular, scheduled nutritional assessments on treatment outcomes and quality of life deserves further exploration in pediatric patients.

## Data Availability

The raw data supporting the conclusions of this article will be made available by the authors, without undue reservation.
